# Fluctuating selection: the perpetual renewal of adaptation in variable environments

**DOI:** 10.1098/rstb.2009.0150

**Published:** 2010-01-12

**Authors:** Graham Bell

**Affiliations:** Department of Biology, McGill University, 1205 Avenue Docteur Penfield, Montreal, Quebec H3A 1B1, Canada

**Keywords:** natural selection, fluctuating selection, selection coefficient, environmental variation, infinitesimal model, oligogenic model

## Abstract

Darwin insisted that evolutionary change occurs very slowly over long periods of time, and this gradualist view was accepted by his supporters and incorporated into the infinitesimal model of quantitative genetics developed by R. A. Fisher and others. It dominated the first century of evolutionary biology, but has been challenged in more recent years both by field surveys demonstrating strong selection in natural populations and by quantitative trait loci and genomic studies, indicating that adaptation is often attributable to mutations in a few genes. The prevalence of strong selection seems inconsistent, however, with the high heritability often observed in natural populations, and with the claim that the amount of morphological change in contemporary and fossil lineages is independent of elapsed time. I argue that these discrepancies are resolved by realistic accounts of environmental and evolutionary changes. First, the physical and biotic environment varies on all time-scales, leading to an indefinite increase in environmental variance over time. Secondly, the intensity and direction of natural selection are also likely to fluctuate over time, leading to an indefinite increase in phenotypic variance in any given evolving lineage. Finally, detailed long-term studies of selection in natural populations demonstrate that selection often changes in direction. I conclude that the traditional gradualist scheme of weak selection acting on polygenic variation should be supplemented by the view that adaptation is often based on oligogenic variation exposed to commonplace, strong, fluctuating natural selection.

## 1. The tradition of gradualism

The central problem of evolutionary biology is to explain how adaptation to particular environmental conditions evolves through natural selection. For almost the whole history of the field, the prevailing view has been that selection is weak and can be effective only when it acts over very long periods of time.

That natural selection always acts with extreme slowness I fully admit.

(Darwin 1859, p. 121)

Thus we come to the conclusion that Darwin was right in regarding transformation as taking place by minute steps, which, if useful, are augmented in the course of innumerable generations …

(Weismann 1909, p. 24)

It must never be forgotten that a very slight advantage will in the course of generations come to predominate.

(Haldane & Huxley 1927, p. 222)

… the extreme slowness of the change revealed whenever we trace evolution in action.

(Wells *et al.* 1934, p. 207)

For ordinary natural selection involving a simple dominant with a selective advantage of 1 in 1000 … it will take nearly 5000 generations to increase the proportion of the dominant from 1 to 50% …

(Huxley 1942, p. 57)

Such calculations are extremely rough, but they suggest the remarkably small magnitude of the selective ‘forces’ which are at work if natural selection is largely responsible for evolution, and the extreme difficulty of detecting them in action.

(Haldane 1949, p. 56)

These estimates of the cost of evolution lead to the conclusion that evolutionary change is normally an exceedingly slow process.

(Mayr 1966, p. 259)

There were few dissentient voices. A remarkable early example was A. R. Wallace, the acolyte of Darwin:

Mr Darwin was rather inclined to exaggerate the necessary slowness of the action of natural selection; but with the knowledge we now possess of the great amount and range of individual variation, there seems no difficulty in an amount of change, quite equivalent to that which usually distinguishes allied species, sometimes taking place in less than a century, should any rapid change in conditions necessitate an equally rapid adaptation.

(Wallace 1889, p. 125)

His was a lone voice. The insistence of Darwin, and his successors, on the prevalence of weak selection operating over long periods of time dominated evolutionary biology for over a century, and inflicted two serious wounds on the development of the field. The first was that attempts to observe natural selection in action would be futile because of the extreme slowness of change. The second was that experiments were out of the question, as no appreciable change could occur within the lifetime of the experimenter. There were brilliant early efforts in both fieldwork (Weldon 1901) and experimentation (Dallinger 1887), but they led nowhere. Field studies were taken over by ecology and laboratory experiments by transmission genetics. Evolution itself became a system of interpretation, almost philosophical in character, operating at the margins of the new disciplines, and promising more than it could perform. A book published to mark the centenary of the publication of the theory of natural selection (Barnett 1958) included chapters on animal breeding, classification, the fossil record, embryology and so forth, but includes no treatment of natural selection in the field or in the laboratory.

This extreme gradualism was codified by Fisher (1930) and others as the infinitesimal model of population genetics. Quantitative characters are taken to be governed by an effectively infinite number of loci bearing alleles of infinitesimal effect. Consequently, character state can be modified by selection with no appreciable change in gene frequencies. This is a convenient calculating device that enables a mathematical theory of quantitative genetics to be built on normal distribution theory (Bulmer 1985, ch. 9). It is not a realistic description of real populations, of course, but it is usually taken to be approximately correct, insofar as the state of a quantitative character is usually governed by a large number of loci each of small effect. Hence, most evolutionary change within a lineage will involve the successive substitution of alleles of small effect. The common-sense justification for this is that any substantial alteration of character state in a well-adapted population is almost certain to be deleterious, whereas very small alterations will often be beneficial (Fisher 1930, ch. 2). The gradual modification of characters in fossil time-series is attributed to stabilizing selection (acting against extreme phenotypes) around an optimal value that changes slowly over time so as to generate very weak but consistent directional selection. In this way, the mathematical theory of population genetics that was developed during the twentieth century formalized and reinforced the gradualist foundation laid down in the nineteenth century.

## 2. The oligogenic model

The renaissance of evolutionary biology as an empirical and experimental science began in the 1950s, from a variety of sources. They included the Oxford school of ecological genetics, and especially the careful fieldwork of Cain and his colleagues (Cain & Shepherd 1954), the analysis of chromosome polymorphism in *Drosophila* by Dobzhansky and colleagues (Dobzhansky 1948) and the artificial selection experiments on *Drosophila* at Leeds and Edinburgh (Clayton & Robertson 1957). By the mid-1980s, the number of field studies had become large enough for Endler (1986) to review the field and conclude that directional selection in natural populations was commonplace and often strong. At about the same time, the emphasis of field research programmes was shifting from measuring the frequencies of discrete morphs to estimating the relationship between the state of continuous characters and fitness components. When this field became mature enough to be reviewed, the results reinforced the conclusions based on polymorphism (Hendry & Kinnison 1999; Kingsolver *et al*. 2001). Moreover, laboratory selection experiments had demonstrated that selection of comparable magnitude to that reported in many field studies was capable of driving mean character state far beyond the limit of variation expressed by the ancestral population within a few dozen generations (Barton & Keightley 2002). There are some reasons to believe that published studies tend to overestimate the average strength of selection: for example, research is more likely to be directed towards situations where selection is more likely to occur, such as newly available habitats and disturbed sites, and positive results may be more likely to be written up and published. Most studies also measure only a single component of fitness, rather than the overall relative rate of increase (Hereford *et al*. 2004). Nevertheless, the field has shifted decisively from supposing that selection coefficients are almost always a fraction of 1 per cent, to recognizing that selection coefficients of 10 per cent are not unusual.

In recent years, we have also learned more about the genetic basis of adaptation. Theoretical work has established that the order in which beneficial alleles are substituted depends on their effects, because alleles of small effect, even though more numerous, are very likely to be lost by drift soon after their appearance. Hence, the bulk of adaptation to new conditions may be attributable to a few mutations of large effect (Orr 2003). This has been confirmed by experimental studies demonstrating that the first beneficial mutations to be fixed have large effects on fitness (Barrett *et al*. 2006) and that adaptation to new conditions typically involves large initial increments in fitness (Dykhuizen & Hartl 1981; Notley-McRobb & Ferenci 1999; Holder & Bull 2001). The genetic analysis of quantitative characters has shown that a few loci may contribute a large fraction of the genetic variance (Kearsey & Farquhar 1998). This conclusion has been disputed because limited studies are capable of detecting only major genes, whereas more extensive surveys often detect a large number of genes of smaller effect (Bost *et al*. 2001). It may well be that quantitative characters are often affected by hundreds or thousands of loci. Regardless of the number of loci that may affect a character, however, the important result from an evolutionary standpoint is that beneficial alleles of large effect exist, and these are most likely to be those that are primarily responsible for adaptation. They may be initially absent from the population, and arise by mutation at rate *u*; or they may already be present, held in mutation-selection equilibrium at a frequency directly proportional to *u*. The oligogenic model applies equally to selection acting on standing genetic variation, and to selection acting on novel mutations, as the source of adaptation.

For example, the evolution of crop plants through artificial selection has hinged on mutations in a few major genes, such as those involved in modifying the structure of the maize plant and the maize cob (Doebley 2004). In more natural situations, classic examples of evolutionary change, such as beak shape and size in Darwin's finches (Abzhanov *et al*. 2004) and body armour and spines in sticklebacks (Colosimo *et al*. 2005), are also based largely on mutations of large effect in one or two genes. Even differences between newly diverged species may often involve changes in very few genes (Orr 2001).

For these reasons, the infinitesimal model has been supplemented, and to some extent replaced, during the past decade by an oligogenic model of adaptation. Rapid adaptation is often driven by strong selection acting on oligogenic variation. I have surveyed the evidence for this in greater detail elsewhere (Bell 2008). There will be many examples, no doubt, where characters evolve slowly over long periods of time through weak selection acting on alleles at many loci. There is no longer any compelling reason to suppose that this is the normal course of adaptation, however, and a great deal of empirical evidence to suspect that it is not. At the same time, however, there are two serious obstacles to accepting the oligogenic model as a general account of adaptation.

In the first place, estimates of heritability in natural populations are often high, with an average of about 0.5 (Weigensberg & Roff 1996). This seems difficult to reconcile with the conclusion that selection is commonplace and often strong. Even fitness components, which should be exceptionally responsive to selection, usually display quite high levels of genetic variance. This might be consistent with the oligogenic model if relative fitness varies in space, between the different habitats occupied by a species. Strong divergent selection may then protect genetic variance, provided that dispersal is not too high, that habitats do not vary much in productivity and that the number of individuals maintained within each habitat is fixed.

Secondly, estimated rates of evolution fall in proportion to the length of time over which they are estimated. The rate of evolution *R* (in Darwins) is related to elapsed time *t* as *R* = *at*^*z*^, with *z* ≈ −1, for times of up to 10^7^ generations (Gingerich 1983). This is in a sense attributable to the negative autocorrelation of rate and time, such that if the absolute difference between final and initial character states is independent of the elapsed time (*r*^2^ = 0), the rate is strongly correlated (*r*^2^ ≈ 0.5), with the slope of the log–log regression being *z* ≈ −1. Hence, the Gingerich rule implies that the corresponding power law for the absolute difference in character state has *z*_D_ ≈ 0 (Estes & Arnold 2007, fig. 4B). Kinnison & Hendry (2001) and Hendry *et al*. (2008) confirmed this result for examples of recent evolution over periods of 10–300 generations, while Bone & Farres (2001) reported similar results for plants. This implies that the amount of evolutionary change (absolute difference in mean character state between two points in time) is independent of the time over which it is measured, or nearly so. The rates of selection characteristic of contemporary populations do not extend into past times, but are consistently diluted so as to maintain more or less the same average amount of change. This remarkable observation seems to confirm that stabilizing selection around a very slowly moving optimum governs long-term evolutionary change. Estes & Arnold (2007) evaluated several different kinds of model and found that the best fit to the Gingerich rule occurred with a displaced-optimum model, in which conditions remain constant for long periods of time, but occasionally and abruptly change. Spatial variation in selection pressure does not provide a convincing alternative: if selection varies in space, the relative productivity of habitats must remain almost the same over long periods of time. Hence, the oligogenic model, with variable selection, appears to require the same unrealistic level of environmental stability as the infinitesimal model.

The only way in which the oligogenic model can be made consistent with high levels of heritability and long-term stasis is to show that selection fluctuates strongly over time, often changing in direction. This could retard the loss of genetic variation, with minimal long-term directional change. It is a radical departure from traditional accounts of adaptation, however, that must satisfy at least three onerous conditions. First, it must be based on an empirically verified theory of environmental change. Secondly, it must be consistent with the Gingerich rule that rates of evolution vary inversely with elapsed time. Thirdly, it must be supported by estimates of selection coefficients, or other parameters such as selection differentials or intensities directly related to selection coefficients, in contemporary populations. In the rest of this article, I shall investigate these three conditions.

## 3. A multi-scale theory of the environment

Natural time-series very commonly have the property that the contribution *C* made by a category of events is inversely proportional to its frequency *f*, such that *C*(*f*) = *f*^−*γ*^ with 0 < **γ** < 2 (Steele 1985; Halley 1996). The two extreme cases are white noise, which has **γ** = 0, and a random walk, or Brownian motion, which has **γ** = 2. Most situations will be intermediate, with **γ** ≈ 1 (a reddened spectrum, or ‘pink noise’, from the optical analogy). Suppose that the frequency *f*_M_ of the category of events of average magnitude *M* declines exponentially with increasing *M* as *f*_M_ = *f*_0_ exp(−**γ*M*), where **γ** now expresses how rapidly the frequency of events falls off with the severity of their effects, while *f*_0_ represents the shortest interval recognized in a survey. The average magnitude of events that occur with frequency *f*_M_ is then *M* = −(1/**γ**) ln(*f*_M_/*f*_0_). We can use this result to calculate the state of the environment at any particular time as the sum of the effects of events that happened in the past, on all time-scales. The fundamental property of this multi-scale model of the environment is most simply depicted from the environmental variance associated with the lapse of time between *t*_1_ and *t*_2_, which is Var(*E*) = ½(*x*_1_ − *x*_2_)^2^, where *x*_*t*_ is the value of an environmental factor at time *t*. This environmental variance increases approximately with the logarithm of the length of the time-series, or with elapsed time *T*, Var(*E*) = *aT*^*z*^. The spectral and power-law approaches are equivalent, with *z* = 0 corresponding to white noise, and *z* = 1 corresponding to Brownian motion. Both formulations imply that variance will continue to increase indefinitely with the length of the time-series, albeit at a decreasing rate.

Physical variation often follows a power law very closely. For example, Koscielny-Bunde *et al*. (1998) analysed deviations of daily maximum temperature from their seasonal average values, estimated with very voluminous and exact data taken at weather stations around the world over a period of more than a century. They found that plots of ln variance on ln elapsed time are almost perfectly linear with **γ** = 0.7 (*z* = 0.35), which they suggest constitutes a ‘universal persistence law’. Vasseur & Yodzis (2004) reviewed datasets for several climatic variables and found the overall average for about 100 historical surveys of mean, maximum and minimum temperature to be about **γ** = 0.7, with precipitation and other factors having lower values. Pelletier (1997) found **γ** ≈ 0.5 (*z* = 0.25) from ice-core samples for elapsed times between one month and 2000 years, with some evidence of larger values for longer and shorter time periods.

This environmental variability could drive changes in the growth and abundance of populations. Pimm & Redfearn (1988) showed that ln abundance *N* of animals also follows a power law. As the ln variance of ln *N* is equivalent to ln [*N*(*t* + 1)/*N*(*t*)], this implies that the variance of realized growth rate increases indefinitely over time. Inchausti & Halley (2002) used the extensive records held in the Global Population Dynamics Database to obtain a median value of *z* = 0.36 across more than 500 long-term datasets. The distribution of *z* showed a mode at small values of 0–0.3 and declined in a roughly exponential manner for larger values. The distribution of **γ** was roughly symmetrical around a mean of 1.0 (*z* = 0.5).

Species may continually fluctuate in abundance through inherent dynamic processes, through forcing by the physical environment or through Red Queen interactions with other species. In any case, fluctuations in the abundance of competitors, parasites and hosts, and predators and prey will in turn impose selection on a target population. This selection may be intense. Many of the classic accounts of selection in natural populations, such as *Cepaea*, *Gasterosteus* and *Geospiza*, discussed below, involve biotic interactions, often modulated by physical factors. Many genes that have a strong genomic signature of strong directional selection govern host–pathogen interactions (Endo *et al*. 1996).

## 4. Adaptation in multi-scale environments

In brief, the physical and biotic conditions of growth vary on all time-scales, with rarer events of greater magnitude making a progressively smaller contribution to the overall variance. Because the environment changes on all time-scales, allele frequencies will also show trends on all scales of calendar time. At the shortest scales, there is strong fluctuating selection that can cause appreciable shifts in allele frequency within a generation, or a few generations. Longer term changes in conditions will create fluctuating selection over longer periods, which if the period is long enough will be perceived as a directional trend. Long-term evolution is usually depicted in two alternative ways: either as a gradual change driven by chronic weak selection or as an abrupt change following a long interval of stasis. I am presenting here a third interpretation that seems to me more consistent with observations of natural selection in open populations: selection is generally rather strong and fluctuates on all time-scales such that abrupt changes can occur over short periods of time and gradual directional change occurs over long periods of time. This process is brought to a halt when the species becomes extinct, at which point it expresses directional change corresponding to the long-term environmental change on the time-scale of its longevity. This is not a new theory: for example, Stanley & Yang (1987) found that morphological characters in fossil series of bivalves fluctuated strongly over time with little directional trend, which they attributed to ‘zigzag selection’. However, it has not yet become established as the usual mode of evolution at all time-scales, although Leroi (2000) takes a very similar view in arguing that the scale independence of adaptive change constitutes a basic evolutionary principle.

This process can be modelled by coupling a multi-scale model of environmental change with an evolutionary model. In the environmental model, any particular category of event occurs at intervals of some given number of years, and during this interval the event continues to have the same effect on conditions of growth. More extreme events occur at longer intervals. An event in the category of events that occur every year will be an exponential random variable with some small average value; an event in the category that occur every 2 years will likewise be exponentially distributed but with somewhat greater average, and so forth. The current state of the environment is then the sum of the effects of events in all categories. Its absolute value is largely determined by rare events of a large magnitude, whereas its variation from year to year is usually attributable to smaller and more frequent kinds of events. Conditions will thereby change on all time-scales, and will fluctuate around an overall trend, no matter what period of time is chosen for a survey. This generates an increase in environmental variance over time that continues indefinitely.

The evolutionary model simulates a population of *N* diploid individuals bearing a given number of loci affecting a quantitative character, to which the environmental variation **σ**^2^_E_ also contributes. New genetic variation is contributed by mutation to the average effect at each locus at rate *u* per locus per replication. The current state of the environment defines the optimal character state, with the fitness of an individual determined by the distance of its phenotype from the optimum, using a Gaussian function of width **ω**^2^. Individuals produce offspring in proportion to their fitness. The evolving population will then track the changing environment, always lagging somewhat behind. Its dynamics will be governed mainly by the mutation supply rate, the heritability and the strength of selection, which depend on *N*, *u*, **σ**^2^_E_ and **ω**^2^. Any result may be obtained, including extinction, but biologically reasonable parameter values will satisfy the following criteria:

- the mutation supply rate *Nu* < 1 per generation (if we are interested primarily in large multicellular organisms),
- the average distance between the population mean and the optimum should be about 1 phenotypic standard deviation (Estes & Arnold 2007, fig. 8; Bell 2008, p. 164),
- the coefficient of variation should be between 0.05 and 0.2 (for morphological characters) (Simpson *et al*. 1960; Mouse Phenome Project 2008),
- the width of the fitness function **ω**^2^ ≃ 10–40 (Estes & Arnold 2007, p. 236),
- the average heritability should not be far from 0.5 (Weigensberg & Roff 1996),
- the exponent *z* of the power law relating environmental variance to elapsed time should not be far from 0.5 (Vasseur & Yodzis 2004), and
- and mean fitness should be adequate to perpetuate the population at all times.

These together ensure that the simulation is biologically realistic.

**Figure 1.**
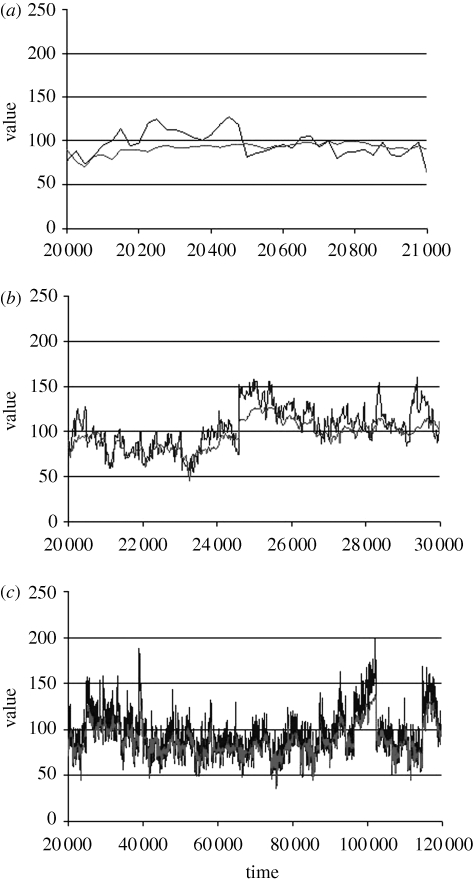
Simulated evolution in a multi-scale environment. Character state is governed by alleles with additive effects at 10 loci. Mutation creates a new allele with an exponentially distributed random effect with mean 1: the mutation rate is 0.001 per locus per generation. Environmental variation adds a random normal deviate to the character state of each zygote with mean 0 and variance **σ**^2^_E_ = 50. There is random gamete fusion and a single crossover with probability 0.5 at each meiosis. The population consists of 500 diploid individuals, of whom 100 are selected at random in each generation, each with probability proportional to its fitness. The width of the fitness function is **ω**^2^ = 25. Over the 10^5^ generations shown in the figure, the realized population variables were: average distance of population mean from optimum = 1.13 **σ**_P_; coefficient of variation = 0.13; average heritability = 0.63; *z*_E_ = 0.25; *z*_P_ = 0.46. The three panels show the population mean tracking the optimum over different time-scales, plotted at 25-generation intervals. (*a*) 1000 generations, (*b*) 10 000 generations, and (*c*) 100 000 generations. Thick line, optimum; thin line, mean.

An example of a simulation that meets these criteria is shown in figure 1, which shows the pattern of environmental change at different time-scales and the response of the mean character state. I have not yet attempted an extensive exploration of how parameter values affect outcomes, but simulations following the restrictions listed above have always produced qualitatively similar results consistent with the conclusions drawn here. We can now ask how the amount of evolutionary change corresponds with observed values by computing the exponent *z*_P_ of the power law for the increase in phenotypic variance through time. The corresponding power law for the absolute difference in character values has *z*_D_ = *z*_P_/2. The environment itself, and hence the optimal character state, follows a power law with exponent *z*_E_, which will vary by chance among simulations between about 0.2 and 0.7. If the population were able to track the changing environment precisely, through intense selection and high mutation supply rate, the mean phenotype would always correspond with the optimum, so that *z*_P_ = *z*_E_. In practice, the population always lags behind the fluctuating optimum because selection can act only by modifying the genotypic distribution of the previous generation. The consequence of this lag is that genetic change is always more coarse-grained than environmental change, so that *z*_P_ > *z*_E_. As *z*_E_ is substantial, this implies that *z*_D_ will also be substantial, and in practice usually exceeds 0.2. The multi-scale model thus predicts that the amount of evolutionary change will steadily increase with elapsed time, which seems inconsistent with the very extensive data that support *z*_D_ ≈ 0.

There are two ways, however, in which *z*_D_ might be estimated. The first is to use the initial and final values from a large number of independent studies involving different organisms and characters. This is the method used by Gingerich (1983) and by Kinnison & Hendry (2001). The second is to sample a single time-series at random intervals of time. This corresponds to the pattern actually shown by an evolving population, and is the method I have used for analysing the multi-scale model. It would be interesting to estimate *z*_D_ in this way for real time-series. Unfortunately, very few evolutionary time-series have been reported in sufficient detail. The most extensive I have found describes morphological change in a foraminiforan (*Afrobolivina*) from 92 horizons in Late Cretaceous sediments (Reyment 1982). Some less-extensive datasets involve a primate (Clyde & Gingerich 1994), a fish (Bell *et al*. 1985), ammonites (Raup & Crick 1981) and radiolarians (Kellogg & Hays 1975). If the rate of evolution in these studies, calculated from the initial and final character states, is plotted on log–log axes, the result is a well-marked negative regression with a slope of −0.8, which is consistent with the Gingerich rule, considering the meagre amount of data (*n* = 13). If we sample the same time-series at various intervals of time, however, we obtain a quite different result: the phenotypic variance (and hence the absolute difference in character state) tends to increase over time in all cases. The *Afrobolivina* series yields *z*_P_ = 0.51 and hence *z*_D_ = 0.25. The other studies yield generally larger values for *z*_P_ (table 1). The data are too scanty to rely on for precise estimates of the pattern of evolutionary change in morphological characters, but they certainly do not support the hypothesis that *z*_P_ ≈ 0. Kinnison & Hendry (2001, table 2) also found that difference almost always increases over time in much shorter contemporary time-series of evolving populations. It appears that the Gingerich result is a consequence of combining estimates of change from many independent studies, and is not a correct description of the pattern of change within a single evolving population.

**Table 1.** Pattern of long-term morphological change. The variance plot is the regression of log phenotypic variance on log time interval, with slope *z*_D_ and correlation coefficient *r*. Spatial scale of each survey is given as extent (total time interval, My) and grain (mean sampling interval, My).

organism	age	survey			variance plot		
		extent	grain	character	*z*_D_	*r*	authority
primate, *Cantius*	Eocene	1.6	0.04	lower molar length	1.10	0.49	Clyde & Gingerich (1994)
fish, *Gasterosteus*	Miocene	0.1	0.005	body length	0.22	0.10	Bell *et al*. (1985)
				anal fin rays	0.68	0.12	
				dorsal fin rays	1.19	0.51	
				post-pterygiophore	0.60	0.28	
ammonite, *Anakosmoceras*	Jurassic	1.4	0.1	shell diameter	0.66	0.2	Raup & Crick (1981)
ammonite, *Zugokosmoceras*				shell diameter	1.12	0.39	
ammonite, *Spinikosmoceras*				outer rib size	0.46	0.16	
ammonite, *Kosmoceras*				outer rib size	1.01	0.39	
radiolarian, *Calocycletta*	Miocene	5.75	0.36	thorax width	1.31	0.47	Kellogg & Hays (1975)
radiolarian, *Pterocanium*	Pliocene	3.9	0.23	thorax length	1.68	0.64	
radiolarian, *Pseudocubus*	Pliocene	1.3	0.08	thorax width	1.03	0.38	
foraminiferan, *Afrobolivina*	Cretaceous	0.5	0.005	pre-loculus diameter	0.75	0.42	Reyment (1982)

Morphological change in fossil time-series has often been interpreted as a random walk. For example, Hunt (2006) interprets the Cantius data (table 1) in terms of a random walk. Estes & Arnold (2007) found that a random walk of the optimum did not fit the Gingerich data, primarily because it predicted a substantial increase in absolute difference over time. The random walk is a special case of the multi-scale model with *z*_E_ = 1, which is indeed inconsistent with single estimates of divergence from many independent surveys. However, more realistic models with 0 < *z*_E_ < 1 do successfully fit values sampled from within a single time-series. The displaced-optimum model of Estes & Arnold (2007) is also a special case of the multi-scale model. As time increases, the probability that the interval includes both environmental states increases; hence, the environmental variance increases steeply (*z*_E_ > 1). For values greater than the displacement interval, however, all comparisons include both environmental states, and the variance is zero. This keeps the long-term divergence, estimated from independent surveys, close to zero. Clegg *et al*. (2008) interpreted morphological change over 30 years in an island population of the passerine bird *Zosterops* in terms of fluctuating selection governed by a displaced-optimum model. A multi-scale model shows only a slight increase in variance over time: for adult wing length, for example, *z*_P_ = 0.16. Hunt *et al*. (2008) describe a convincing example of evolution towards a displaced optimum for a freshwater population of sticklebacks (*Gasterosteus*), in which armour and pelvic spines were consistently reduced over a period of several thousand years. Indeed, the displaced-optimum model is likely to be successful in segments of a multi-scale model following some exceptionally wide environmental excursion. Over longer periods of time, however, it is likely to break down, as in the longer term *Gasterosteus* series (Bell *et al*. 1985; table 1), and indeed, in this case, the initially heavily armoured type itself replaced a more lightly armoured type.

The multi-scale model of fluctuating selection is more general than the random-walk or displaced-optimum models, generates a correct description of environmental change and is consistent with the little we know quantitatively about the pattern of change in evolving populations. This provides only weak support for the model, however, as many other theories might fit the data; for example, that morphology is directly influenced by the state of the environment. What direct evidence is there of the strong fluctuations in selection that the model requires?

## 5. Fluctuating selection

The crucial parameter is the standard deviation of the selection coefficient **σ**_*s*_, or equivalently of the intensity of selection **σ**_*i*_, over time. If this is large relative to the mean, there are likely to be large changes in magnitude and frequent reversals in the direction of selection. It could be calculated directly, from demographic surveys of survival and fecundity, or indirectly, through surveys of genotype frequencies. Either method requires extensive time-series, which are even less readily available for contemporary populations than for fossils. In practice, most studies rely exclusively on frequencies; the capture–recapture study of *Cepaea*, described below, is the only exception I am aware of. Frequencies may vary over time through drift, fluctuating selection, immigration or sampling error. Few studies estimate or eliminate each of these processes, so attributing all changes in frequency to fluctuating selection will give only an upper bound on **σ**_*s*_.

### (a) Panaxia

Although Fisher developed the infinitesimal theory, he was also the author of the first paper to estimate variation in the direction and intensity of selection over time. Fisher & Ford (1947) recorded the frequency of variants of the moth *Panaxia* (*Callimorpha*) *dominula* over 8 years in an isolated population of a few thousands of adults. The variation involves the pattern of spotting on the wings, which is governed by a single partially dominant gene, *medionigra*, so that allele frequencies can be obtained from the phenotypic survey. They found that allele frequencies fluctuated irregularly from year to year, to a greater extent than could be accounted for by drift and sampling error; immigration was held to be negligible. This gave rise to a famous controversy (Wright 1948) that continues to the present day. The *Panaxia* survey has also continued, so that records now extend over 60 years (with a 10-year gap). During this time, the frequency of *medionigra* has consistently declined to a low but apparently stable value of a few per cent (reviewed by Cook & Jones 1996; O'Hara 2005). The mean selection coefficient is −0.103, with **σ**_*s*_ = 0.075. The number of variant individuals was often very small (less than 10), especially in later years, so the estimates are rather imprecise. Moreover, the agent of selection is unknown. This is, however, the longest series of records from a natural population that is currently available.

### (b) Cepaea

**Figure 2.**
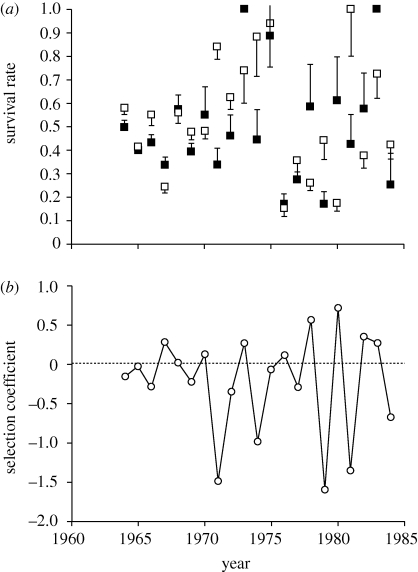
Fluctuating selection in *Cepaea*. Estimates of survival rates for brown (black squares) and non-brown (white squares) individuals from mark-recapture surveys. The bars are 1 s.e. (adapted from Cain *et al*. 1990). (*a*) Survival rates and (*b*) fluctuation of the selection coefficient.

Cain *et al*. (1990) surveyed a population of the helicid snail *Cepaea nemoralis* living in downland in southern England over a period of 23 consecutive years. Each year, they captured and marked adult snails, and estimated annual survival rates from the frequency of recaptured snails in the following year. The snails mature at 2 years of age, after which the average annual survival is approximately 50 per cent. Hence, the survey gave estimates of one component of fitness, adult survival, over about half the adult lifespan, for a combined sample size of about 10 000 individuals. These estimates were reported separately for morphs differing in the ground colour and banding pattern of the shell, characters that are known to be determined primarily by alleles at two linked loci. The example given in figure 2 shows the survival of brown (determined by a single dominant allele) relative to non-brown individuals. There is no average effect of the allele on relative survival, nor any trend over time, but there are wide fluctuations in relative survival during the survey period. In years where there is a significant (*p* < 0.05) difference between brown and non-brown phenotypes, brown has lower survival in six cases and higher survival in two cases. The selection coefficient calculated from survival rates has **σ**_*s*_ = 0.65; estimates for other morphs are **σ**_*s*_ = 0.76 for pink versus yellow, and **σ**_*s*_ = 0.56 for banded versus unbanded. These are very large values, perhaps because they include an unknown, but probably substantial, contribution from sampling error.

### (c) Gasterosteus

Reimchen & Nosil (2002) estimated the intensity of selection acting on spine number in a population of three-spined sticklebacks (*Gasterosteus aculeatus*) by following cohorts over time. The spines provide protection against gape-limited predators, such as diving birds, but may facilitate predation by grappling predators, such as dragonfly larvae. The combined sample size was about 14 000 individuals collected over 12 years, with some missing data. The largest dataset (winter samples of adult males, over 11 years) gave an average value of *i* = 0.03, with **σ**_*i*_ = 0.21. Estimates for females, younger life stages and summer conditions yielded an average value of **σ**_*i*_ = 0.30. If the estimate for a given series (sex, life stage and season) is plotted on the mean sample size *n*, it is reasonably well-fitted by the function **σ**_*i*_ = *L* + exp(−*kn*), where *L* is the limiting value of the estimate of **σ**_*i*_ in the very large sample. The best fit is given by *L* = 0.18, which is close to the value for adult males in winter (*n* = 220). There is only a modest range of sample sizes (133–220), however, and not much difference between *L* = 0.18 (*r*^2^ = 0.57) and *L* = 0 (*r*^2^ = 0.52). Reimchen & Nosil (2002) argue that changes in the direction of selection may be associated with shifts in feeding behaviour between limnetic foraging for zooplankton, when the fish are exposed to predation by birds, and littoral foraging for macrobenthos, when they are attacked by dragonfly larvae.

### (d) Daphnia

Lynch (1987) conducted a survey of isozyme genotypes in populations of the cladoceran *Daphnia* that were arguably too large for drift to be important, and too isolated for any substantial level of immigration. He developed sampling theory for estimating variation in the selection coefficient independently of sampling error. Samples were taken at intervals during the growing period, so the mean and variance of the selection coefficient are estimated for the five to 20 vegetative generations occurring per year. His own data yielded an estimate of **σ**_*s*_ = 0.195 for homozygotes, and he analysed other *Daphnia* data from the literature that gave an average value of **σ**_*s*_ = 0.11. These are probably the best estimates of **σ**_*s*_ currently available.

### (e) Ficedula

Tarsus length was correlated with viability in a pedigree population of pied flycatchers, *Ficedula hypoleuca*, that was followed for 18 years (Kruuk *et al*. 2001). The selection differential fluctuated over time, with **σ**_*s*_ = 0.13 for phenotypic values and **σ**_*s*_ = 0.14 for breeding values. It was consistently positive throughout the study period, however, with no convincing evidence for a reversal of sign.

### (f) Geospiza

The best-known example of fluctuating selection driven by a known selective agent in a natural population is the change of beak shape in the large ground finch (Darwin's finch) *Geospiza fortis* on the island of Daphne Major in the Galapagos. A prolonged drought in 1976–1977 caused a change in the composition of the vegetation by favouring plants with large, tough-shelled seeds. These could be consumed only by finches with unusually large and powerful beaks, and between 1976 and 1978, beak depth increased at a rate of 26.1 kDar (Boag & Grant 1981). Heavy rain in 1983 reversed the trend in the vegetation by favouring plants with smaller, softer seeds that germinated more readily and thereby favoured birds with smaller beaks that were more adept at processing them (Gibbs & Grant 1987). Within a few years, the response to reversed selection at a rate of 8.8 kDar had more or less restored the status quo (Grant & Grant 1995).

### (g) Ovis

Charbonnel & Pemberton (2005) detected fluctuating selection at an MHC locus during a 13-year survey of a population of feral sheep, perhaps driven by interactions with parasitic nematodes.

### (h) Diaptomus

Hairston & Dillon (1990) surveyed a pond population of the copepod *Diaptomus sanguineus* over 10 years, finding that selection acting on the timing of diapause shifted between years according to the intensity of predation by fish. From their estimates of response to selection and heritability, the mean selection coefficient was *s* = 0.017 with **σ**_*s*_ = 0.097.

### (i) Hordeum

The barley Composite Cross V was founded by intercrossing 30 barley cultivars from different regions of the world and subsequently propagated for many years without conscious selection under standard agricultural conditions in California. Clegg *et al*. (1978) grew stored seed from populations 10 generations apart in uniform conditions and estimated the fitnesses of single-locus isozyme genotypes. The standard deviation of *s* between populations for homozygotes was **σ**_*s*_ = 0.087, again an upper bound because the populations being compared are 10 generations apart.

### (j) Euphydryas

Mueller *et al*. (1985) scored polymorphic enzyme loci in two populations of the butterfly *Euphydryas* over 8 years. They concluded that drift could not account for the observed variation in frequency at three of these loci in one of the populations. I have reanalysed their data by Lynch's method, excluding two loci with little variation and 1 year in one population when sample size was very small. There seems to be a single case, involving a hexokinase locus, in which fluctuations in frequency are too large to be explained in terms of drift and sampling error. It is possible, however, that immigration contributes to changes of frequency in this highly vagile insect.

### (k) Other organisms

There are also several shorter term studies that have reported shifts in the magnitude or direction of selection in consecutive years, including flowering time in *Erythroxylum* (Dominguez & Dirzo 1995), *Carlina* (Rees *et al*. 2004) and *Digitalis* (Sletvold & Grindeland 2007), and germination time in *Collinsia* (Kalisz 1986). These studies show that selection can change direction over short periods of time, but they might easily be matched or outnumbered by others where consistent selection is observed but not emphasized. Populations of *Cepaea* that have been resampled after a lapse of 15–25 years may be essentially unchanged (Arthur *et al*. 1993; Cameron 2001) or may show substantial shifts in morph frequencies (Wall *et al*. 1980; Cowie & Jones 1998; Cook *et al*. 1999).

**Figure 3.**
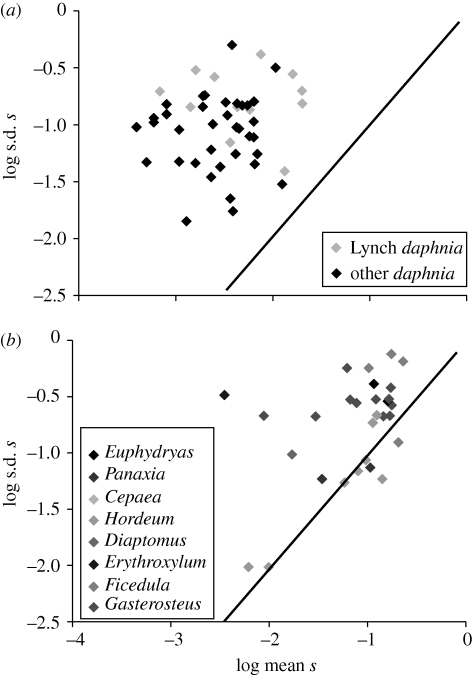
Fluctuating selection in natural populations. The standard deviation of the selection coefficient over time **σ**_*s*_ is plotted against its mean absolute value |*s*|. The solid line is **σ**_*s*_ = |*s*|.

These studies are summarized in figure 3. In most cases, **σ**_*s*_ > |*s*|, implying that selection often reverses in direction. One obvious and important reservation is that estimates of **σ**_*s*_ may be inflated by sampling error. Where an attempt has been made to eliminate this source of variation (as in the *Daphnia* estimates), the result is unaffected, but in other cases sampling error remains a serious concern. A conservative conclusion might be that the limited evidence available for contemporary selection fails to refute the oligogenic model of adaptive evolution.

## 6. Conclusion

The argument made in this article can be briefly stated as follows.

- The physical conditions of life change continually on all time-scales,
- this contributes to the continual change in community composition,
- changes in the physical and biotic conditions of life impose strong directional selection on populations,
- as the agents of selection vary continually over time, the direction of selection often changes, and adaptive walks are often interrupted,
- beneficial alleles of large effect are likely to be the first to be fixed in an adaptive walk, and
- hence, adaptation will often involve alleles of large effect.

Thus, selection in natural populations is commonplace, often strong, fluctuating and oligogenic. I hope that this argument will help to reconcile the oligogenic theory of adaptation, which has emerged over the past decade from empirical work in the field and in the laboratory, with apparently contradictory observations such as the Gingerich rule of long-term stasis. This shift from the infinitesimal model as the prevalent account of the mechanism of adaptation will have profound implications for evolutionary biology, as it becomes an increasingly experimental field. It is not yet firmly established, however. There is some evidence that the extent of evolutionary change increases indefinitely over time, as predicted by a multi-scale model of the environment, and time-series of genotype frequencies suggest that selection often changes direction. The information currently available is inconclusive, however, because the great application that is required to follow populations of snails, fishes or birds over many years has greatly restricted the number of studies that can be used to validate the basis of the multi-scale oligogenic model. This cannot be remedied in the short term: spatial surveys can be greatly extended simply by applying more effort, but the rhythm of temporal surveys cannot be accelerated.

The study of adaptation in the laboratory has been facilitated by the use of microbial systems, which are in effect time machines that make use of the short lifespan of microbes to compress many generations within a short period of calendar time. These experiments have almost all been conducted with single-species cultures living in simple, uniform environments, and we know very little about how populations and communities adapt to complex or changeable conditions of growth (Barrett *et al*. 2005). There is considerable scope for experimentation in more realistic conditions, to determine how adaptation is modulated by complex and changing conditions, involving rivals and enemies. More radically, the same approach could be applied to field experiments involving populations living in a completely natural setting, by developing model systems, for example of yeasts or pseudomonads, that could be inoculated into natural communities and subsequently recovered, identified and typed. An ecologically and genetically well-known microbial system could be used to identify the agents of selection (currently almost unknown) that act under natural conditions of growth, and to determine how their action varies in space and time. Formidable logistical difficulties would have to be overcome for this project to succeed, but it would provide a sound basis for testing the multi-scale oligogenic model of evolution.
